# An Open Letter

**Published:** 1901-09

**Authors:** I. B. Davenport


### Foreign Correspondence.

### AN OPEN LETTER.

Extracts from a postscript to a letter of April 12, 1900, to Dr. W. C. Barrett, chairman of the Foreign Relations Committee of the National Association of Dental Faculties.

Paris, May 20, 1901.

Dear Doctor,—As you did me the compliment to invite my personal opinion on professional matters, I have delayed sending the above letter of April 12, always hoping to be able to add a few impressions.

As I understand the matter, the main object of these foreign boards is to help insure that the D.D.S. degree carries with it a guarantee of professional respectability so far as preparation and course of study is concerned, and, supposing that to be established, I have noticed a great tendency on the part of members of these boards to insist that respectability of the degree ought to carry with it the right to practise anywhere. This, if theoretically true, would never be admitted abroad, and actually is not admitted even at home.

Your circular of December 10, 1900, I most heartily approve, as it clearly limits the power of the boards. A little power is a dangerous thing in the hands of some people, and especially to those unaccustomed to it, and the real object—the general good—may be forgotten in the opportunity to protect private and personal aims.

In general a board which cannot, on account of the personal elements composing it, command at least respect in the country in which it is located cannot be efficient in smoothing out the prejudices against our degrees, but, on the contrary, being itself discredited, throws also discredit upon the institution which it is supposed to represent, as well as upon the products of these institutions.

Nothing tends more to place a board in discredit than the attempt to influence the laws of the country in its own favor. In each country the American dentist finds opposition and difficulties peculiar to it, and in meeting these many personal elements arise which are too apt to influence the action and attitude of the board.

It may be gratifying to a man who finds himself rather isolated in a foreign country and pressed by hostile competitors, to be able to show that he is backed by the whole dental educational system of America, through being a member of the Advisory Board of the Foreign Relations Committee of the National Association of Dental Faculties of the United States of America, and he can drop a hint to that effect, and of vague possibilities of evoking retaliatory measures.

Some of the boards have done good work in the way of showing up bogus diplomas and bogus institutions, but for Heaven's sake "save us from our friends" if we must continue to read these exposures. We have had enough. These things are exploited to our discredit by men who know better. The doings of an admitted bogus institution are made to apply to all our colleges, before legislators and before the public, just as the "Buchanan" affair was made use of by the Doyen (Dr. Bronardel) of the Faculty of Medicine before the Parliamentary Committee when the French Medical and Dental Law of 1892 was being passed, to throw discredit upon all our professional schools.

It is not the *whole* truth some of these foreigners want, it is part of the truth,—just enough to use against us. This is a regular plan, a part of a propaganda to discredit the American dental degree, and why? Because its superiority and value is fully recognized in Europe, to the detriment (they consider) of native talent, and so much so that (while almost no American advertises in Europe) every quack and advertiser of Europe pretends to be American or advertises to practise the American system; and it is this well-recognized superiority of the dentist educated in America that has led unscrupulous men to exploit this good name and reputation for base purposes.

It is well to know one's own faults and weaknesses, but we had better stop advertising them and spend our efforts in correcting them and living them down.

Let foreign governments hunt up their own bogus doctors, and let the American dental colleges give attention to their own standards.

I fear that the giving of these foreign boards an official character is, after all, a mistake, and likely to do more harm than good.

Yours very sincerely,
I. B. Davenport.

